# Quantitative Cryo-TEM Reveals New Structural Details of Doxil-Like PEGylated Liposomal Doxorubicin Formulation

**DOI:** 10.3390/pharmaceutics13010123

**Published:** 2021-01-19

**Authors:** Rickard Nordström, Lin Zhu, Johan Härmark, Yael Levi-Kalisman, Erez Koren, Yechezkel Barenholz, Genia Levinton, Dima Shamrakov

**Affiliations:** 1 Vironova AB, Gävlegatan 22, 113 30 Stockholm, Sweden; rickard.nordstrom@vironova.com (R.N.); lin.zhu@vironova.com (L.Z.); johan.harmark@vironova.com (J.H.); 2 Institute for Life Sciences and The Center for Nanoscience and Nanotechnology, The Hebrew University of Jerusalem, Jerusalem 9190401, Israel; yael.kalisman@mail.huji.ac.il; 3 Laboratory of Membrane and Liposome Research, Hadassah Medical School, The Hebrew University, IMRIC, Jerusalem 9112001, Israel; erezkoren@gmail.com (E.K.); chezyb@ekmd.huji.ac.il (Y.B.); 4 Ayana Pharma Ltd., Hadassah Ein Kerem Campus Biotechnology Park, Jerusalem 9112002, Israel; genia@ayanapharma.com

**Keywords:** SAXS, cryo-TEM, PEGylated liposomal doxorubicin (PLD), dynamic light scattering (DLS), nanoparticle tracking analysis (NTA)

## Abstract

Nano-drugs based on nanoparticles (NP) or on nano-assemblies as carriers of the active pharmaceutical ingredient (API) are often expected to perform better compared to conventional dosage forms. Maximum realization of this potential though requires optimization of multiple physico-chemical, including structural and morphological, parameters. Meaningful distributions of these parameters derived from sufficient populations of individual NPs rather than ensemble distributions are desirable for this task, provided that relevant high-resolution data is available. In this study we demonstrate powerful capabilities of the up-to-date cryogenic transmission electron-microscopy (cryo-TEM) as well as correlations with other techniques abundant in the nano-research milieu. We explored Doxil^®^-like (an anticancer drug and the first FDA-approved nano-drug) (75–100 nm) PEGylated liposomes encapsulating single doxorubicin-sulfate nano-rod-crystals (PLD). These crystals induce liposome sphere-to-ellipsoid deformation. Doxil^®^ was characterized by a multitude of physicochemical methods. We demonstrate, that accompanied by advanced image-analysis means, cryo-TEM can successfully enable the determination of multiple structural parameters of such complex liposomal nano-drugs with an added value of statistically-sound distributions. The latter could not be achieved by most other physicochemical approaches. It seems that cryo-TEM is capable of quantitative description of individual liposome morphological features, including meaningful distributions of all structural elements, with averages that correlate with other physical methods. Here it is demonstrated that such quantitative cryo-TEM analysis is a powerful tool in determining what is the optimal drug to lipid ratio in PLD, which is found to be the drug to lipid ratio existing in Doxil^®^.

## 1. Introduction

Nanomedicine is an emerging discipline which applies opportunities provided by nanotechnology to the healthcare. One of its unique aspects is the use of drugs and imaging agents in the form or as a part of nanoparticles (NP). Numerous nano-drug delivery systems developed for different routes of administration include liposomes, dendrimers, nano-crystals and others [1,2,3]. Liposome-based nano-drugs are considered preferable for parenteral administration, and this trend is believed to last for the next coming years as most nano-drugs currently in clinical studies are also based on nano-liposomes or their related lipid nano-particles [3]. The incentive to use nano-drugs stems from incorporation of active pharmaceutical ingredient (API) or imaging agent as a part of a NP of a specifically engineered composition, size, structure, and morphology. This is expected to better control drug performance with respect to therapeutic or imaging efficiency due to significant impact on pharmacokinetics and biodistribution. For example, using PEGylated NP prolongs API circulation time to a large extent, and, in cases of cancer, inflammation and infection enables more API to reach the disease site due to the porosity of blood vessels concomitantly with less systemic exposure of healthy tissues. This leads to substantial improvement of the therapeutic index of the drug due to the combination of better therapy combined with better patient compliance. However, these expectations, in many cases were not met in full, as, in some cases, the benefit was mainly major reduction in toxicity and side effects which was not followed by the major improvement in therapeutic efficacy [4,5].

There are few reasons why the latter expectations were not met, some of them related to the lack of full understanding of detailed structure and morphology of the nano-drug and its cross-talk with the biological milieu [4,5]. This justifies major efforts dedicated to development of multiple physicochemical characterization methods including those for size, fine structure and morphology, preferably as a distribution of individual particles. With respect to high-resolution structure, X-ray scattering methodologies are currently the techniques of choice as they allow to get structural parameters and dimensions at nanometer resolution [6,7]. However, they only represent ensemble structural parameters as averages of the entire NP population, rather than distributions of individual particles, which are much more relevant to the characterization of the NP as drugs.

In this study we suggest that cryo-TEM accompanied by appropriate advanced image-analysis tools, can successfully complement X-ray scattering in determination of multiple structural parameters of complex liposomal nano-drugs, with the additional benefit of providing quantitative and statistically sound distributions of individual NPs for size, structure and morphology parameters.

An extensive review of current imaging techniques available for characterizing nano-liposome morphology, such as various SEM, TEM, ESEM and AFM approaches, were recently surveyed [8]. Robust methodologies of negative staining and cryo-TEM of liposomes were detailed [9].

We used Doxil^®^-like PEGylated liposomal doxorubicin as a model to demonstrate cryo-TEM capabilities, and, wherever relevant, correlation with other wide-spread characterization techniques. Doxil^®^ (also referred to as Caelyx^®^—European brand name) is the first FDA-approved nano-drug as well as the first FDA-approved liposomal drug [10]. Doxil^®^ is a good example of how nano-technology principles can be applied to a successful anti-cancer drug. Since its approval in November 1995 [10,11,12,13], it is in an extensive clinical use for treating various kinds of cancer [10], with almost 600,000 patients treated until October 2017 (Janssen, Pharmaceutical companies of Johnson & Johnson [14]). Ever since, Doxil^®^ is recognized as the gold standard of an injectable nano-drug and drug delivery system. The three FDA approved generic versions of Doxil^®^ (Sun Pharma (approved 2013), Dr. Reddy’s (approved 2017), and Zydus Cadila (approved 2020) all spent major efforts on the approval which prove the large interest in this drug-product

Doxil^®^ is a PEGylated liposomal doxorubicin (PLD), in which the drug is loaded remotely and actively into the intra-liposome aqueous phase of PEGylated nano (75–100 nm) PEGylated liposomes driven by the chemical engine of the transmembrane ammonium gradient, having the bi-valent sulfate as ammonium and doxorubicin counter anion which stabilizes the loading and leads to its zero-order slow release [10,11,15,16,17,18,19].

The remote loading of doxorubicin results in formation of a twisted rod-like doxorubicin-sulfate crystal inside the intra-liposome aqueous phase, forcing the liposome membrane to stretch from spherical to an ellipsoid, “coffee-bean”-like shape [20].

The physicochemical features of Doxil^®^ and their relevance to its performance in-vivo were studied by a variety of techniques, including different methods of size distribution measurement: wide-angle and small-angle X-ray scattering (SAXS, WAXS) [6,20,21], differential scanning calorimetry (DSC) [22] and others [23]. Most of these methods deal with the ensemble population as-a-whole rather than with individual particles. In contrast, electron microscopy and, especially, cryogenic transmission electron microscopy (cryo-TEM) allows direct observation of individual liposomes and enable presentation of real distributions of any feature being visualized. It was back in 1992 when the “coffee-bean” shape of Doxil^®^ was demonstrated by cryo-TEM [20]. In the same study, small angle X-ray scattering (SAXS) demonstrated the crystalline nature of doxorubicin sulfate rod inside the liposome [20]. However, at that time, the fine structural details of Doxil^®^ could not be resolved due to technical limitations. Three-dimensional (3D) tomography imaging of Doxil^®^ liposomes was attempted [24], yet at low resolution. Recently high-resolution average structure of Doxil^®^ was reconstructed using SAXS [6]. Fine structure of individual Doxil^®^ liposomes, which would enable to construct a real size and morphology distributions, is still missing. This study aims to fill this gap and to gain detailed structure for sufficient number of individual Doxil^®^ liposomes thereby constructing size and morphology (which includes liposome-shape and intra-liposome doxorubicin sulfate nano-rod crystal) distributions at high resolution. In addition, this study demonstrates what is required in order to have a meaningful cryo-TEM as a trustable quantitative tool of the structural analysis of complex NP like Doxil^®^.

This study focuses on a careful processing of high-resolution cryo-TEM images and relevant correlations with dynamic light scattering (DLS), nano-tracking analysis (NTA) and small-angle X-ray scattering (SAXS). First, we determine the minimal number of particles required to achieve meaningful distributions. Then we demonstrate the power of cryo-TEM in achieving size and morphology distributions of a Doxil^®^-like product, including its intra-liposomal doxorubicin-sulfate nano-rod crystal.

## 2. Materials and Methods

### 2.1. Materials

Pharmaceutical-grade doxorubicin HCl was acquired from Sicor Societa Italiana Corticosteroidi s.r.l. Cholesterol, hydrogenated soybean phosphatidylcholine (HSPC) and N-(carbonyl-methoxy-polyethylenglycol-2000)-1,2-distearoyl-glycero-3-phosphoethanolamine sodium (MPEG-DSPE) were purchased from Lipoid GmbH, Ludwigshafen, Germany. 1,2-dioleoyl-3-trimethylammonium propane methane sulfonate salt (DOTAP) was from BioLab Ltd., Jerusalem, Israel. L-Histidine, sucrose, dehydrated ethanol and ammonium sulfate, all pharmaceutical-grade, were from Merck KGaA, Darmstadt, Germany.

NIST-traceable standard polystyrene nanoparticles (NPs) of 75.8 nm diameter (Cat. NT04N) were purchased from Bangs Laboratories, Inc. (Fishers, IN, USA). As specified by the supplier, the size of these NPs was calculated from the diffusion coefficient determined by ultracentrifugation.

### 2.2. Preparation of Liposomes

Doxil^®^-like PEGylated liposomal doxorubicin (PLD) was prepared by Ayana Pharma as described elsewhere [6] with 3.5 mM (2 mg/mL) doxorubicin hydrochloride, of which ~98% were entrapped in the liposomes. Cholesterol, HSPC and MPEG-DSPE in a ratio 1:3:1 (% *w*/*w*) consisted total lipids of 16 mg/mL. Doxorubicin to total lipid ratio (*w*/*w*) of Doxil^®^ is 0.125.

**Figure 1:**
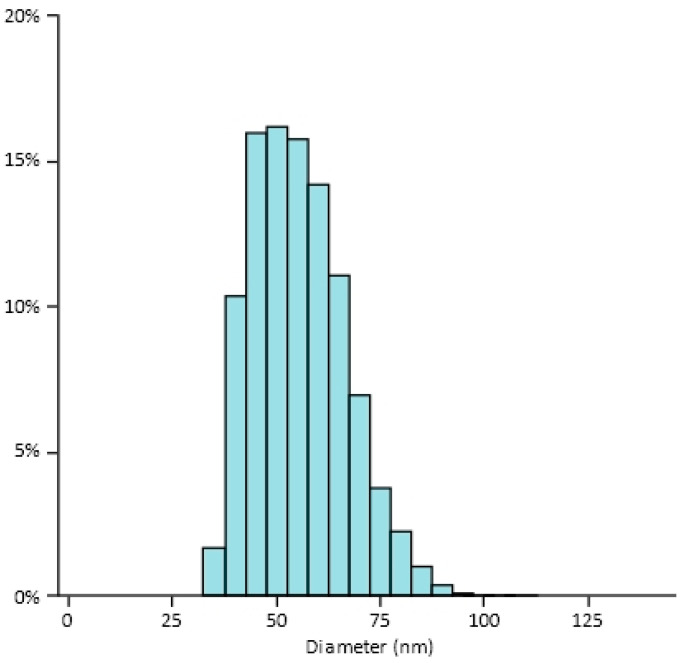
Liposome size distribution measured by cryo-TEM.

In the extrusion process applied, mainly the larger particles are gradually downsized, which results in an asymmetrical size distribution (Figure 1).

PLDs of different doxorubicin to lipid ratio (from 0.00 to 0.38 *w*/*w*) of the same lipid composition and the same lipid concentration were prepared as described for Doxil^®^-like PLD [6].

Similarly, liposomes without MPEG-DSPE were prepared with HSPC and cholesterol in a 3:1% *w*/*w* (~1.5:1 mole) ratio and actively remote loaded with doxorubicin similarly to what is described elsewhere [15]. All PLDs and non-PEGylated doxorubicin remotely loaded nano-liposomes were stored in isotonic 10% sucrose solution buffered to pH 6.5 with 10 mM histidine.

Additionally, liposomes based on DOTAP (instead of HSPC) and cholesterol in a 4:1% *w*/*w* (~2:1 mole) ratio and 6.5 mg/mL of the lipids were prepared, without doxorubicin.

### 2.3. Size Determination by Dynamic Light Scattering (DLS)

Size and size distribution (PDI) were determined by dynamic light scattering (DLS) measured with a Malvern Zetasizer Nano ZS instrument (Malvern, Worcestershire, UK) at an angle of 173°. PLD sample was diluted 1:50 in sterile saline (0.9% NaCl), pre-filtered through a 200 nm pore-membrane.

### 2.4. Nano-Particle Tracking Analysis (NTA)

The count and diameter of liposome were measured by nano-particle tracking analysis (NTA) using Nanosight NS300 instrument of Malvern (Worcestershire, UK). The sample was diluted 1:50,000 (*v*/*v*) with isotonic 0.9% saline filtered through a 20 nm pore Anotop™, filter (Whatman, Germany, ca. 6809–2002). The tracking camera of the instrument has its limitations, which necessitate substantial dilution of the sample. Large dilutions increase the relative contribution of extraneous particulate (<0.2 µm) contaminants, that, in common laboratory practice, are uncontrolled. Therefore, the filtration of the dilution medium was essential to obtain unbiased results.

NTA technique is unique for its capability of direct counting the nanoparticles (~1014 mL^−1^ liposomes in a Doxil^®^-like preparation). Unlike most conventional DLS instruments, it allows to determine particles concentration and provides mode mean in size distributions thus generated.

### 2.5. Cryogenic Transmission Electron Microscopy (Cryo-TEM)

Three TEM instruments were involved in this study.

#### 2.5.1. FEI Tecnai 12 G2 Microscope with Gatan Workstation, Cryo-Holder for Imaging at about −180 °C and Slow Scan Cooled Charge-Coupled Device CCD Camera Gatan 774 (Ilse Katz Institute for Nanoscale Science and Technology, Ben-Gurion University, Beer-Sheva, Israel)

Liposomal samples were vitrified on copper grids coated with a perforated lacey carbon 300 mesh (Lacey substrate, 300 mesh, Ted Pella, Inc., 4595 Mountain Lakes Blvd., Redding, CA, USA). About 3 µL drop was applied to a grid and blotted with a filter paper to form a thin liquid film of solution. Blotted samples were immediately plunged into liquid ethane at just above its freezing point (−183 °C) using either Leica EM GP cryo-preparation station or Vitrobot. The vitrified samples were stored under liquid nitrogen before being transferred to a TEM using a Gatan workstation and cryo-holder for imaging at about −183 °C. The microscope was operated at 120 kV in a low electron dose mode (to reduce radiation damage) and with a few micrometers under-focus to increase phase contrast. The images were recorded on a slow scan cooled charge-coupled device CCD camera (Gatan) supported by the Digital Micrograph software package.

#### 2.5.2. JEM-2100F Microscope (JEOL Ltd. at Department of Bioscience and Nutrition, Karolinska Institutet, Hälsovägen 11, S-141 52 Huddinge, Sweden) Equipped with a Field Emission Gun Operating at 200 kV, Gatan 626 Single Tilt Cryo-Holder and 4 k × 4 k TemCam F415MP Detector (Tietz Video and Image Processing Systems GmbH, Eremitenweg 1, D-82131 Gauting, Germany)

Standard 400 mesh copper grids coated with a Quantifoil^®^ R 2/4 holey carbon film overlaid with a thin continuous carbon film (Quantifoil Micro Tools GmbH, In den Brückenäckern 4, 07751 Großlöbichau, Germany) were used. The grids were hydrophilized using a glow discharger (Peclo EasiGlow, TedPella Inc., 4595 Mountain Lakes Blvd., Redding, CA, USA) and mounted on a vitrification robot (Vitrobot™ Mark I, FEI, Hillsboro, OR, USA). 3 µL of sample were applied onto the grid in the specimen chamber of the vitrification robot, under temperature and humidity-controlled conditions. After an adsorp-tion time of ca. 10 s the grid was blotted-off and subsequently plunge-frozen in liquid ethane.

#### 2.5.3. FEI Tecnai 12 G2 TWIN TEM Operated at 120 kV and Equipped with a Gatan Model 626 Cold Stage (The Center for Nanoscience and Nanotechnology, The Hebrew University of Jerusalem, Jerusalem 9112001, Israel). The Images Were Recorded by a 4 k × 4 k FEI Eagle CCD Camera in Low Dose Mode. TIA (Tecnai Imaging and Analysis) Software Was Used to Record the Images

Samples 3 μL were applied onto a glow-discharged TEM grid (300-mesh Cu grid) coated with a holey carbon film (Lacey substrate, Ted Pella, Ltd., 4595 Mountain Lakes Blvd., Redding, CA, USA). The excess liquid was blotted, and the specimens were vitrified by a rapid plunging into liquid ethane precooled with liquid nitrogen using Vitrobot Mark IV (FEI).

### 2.6. Analysis of Cryo-TEM Images

All the acquired images were uploaded to the Vironova Analyzer Software (Vironova AB, (Gävlegatan 22, 113 30 Stockholm, Sweden). Automatic particle picking was performed on the relevant images, followed by a manual curation step, during which falsely detected particles were removed and undetected particles of interest were added. An independent review of the particle detection was performed in order to limit the number of erroneously identified particles. The data and statistics obtained from the image analysis were extracted and handled in MS Excel (Microsoft Corporation).

## 3. Results and Discussion

Our selection of Doxil-like products as the focus of this study originates in: (i) Doxil^®^ being the gold standard of an injectable drug delivery system; (ii) being in extensive clinical use for more than 20 years; (iii) being a complex system which structural analysis presents a real challenge. Liposomes in Doxil^®^ and its generic versions produced by Sun Pharma and Dr. Reddy’s are not spherical, and their deviation from sphericity is imposed by doxorubicin-sulfate nano-rod crystal present in the intra-liposome aqueous phase. In-depth structural characterization of these intra-liposome drug crystals, which are very important to the PLD performance [25] in-vivo, adds another important dimension to what is known so far. Our studies take the advantage of the fact that these PLDs are well characterized by many physical methods (see introduction above) that describe average physical parameters of the liposome population-as-a-whole. SAXS, for example gives average dimensions of Doxil^®^ membrane and of its intraliposomal doxorubicin-sulfate nano-rod crystal, while DLS provides number and/or volume-weighted liposome whole population size distribution treating the liposomes as spheres. The values obtained are in a reasonable agreement with the cryo-TEM analysis [26]. NTA stands apart as it features possibility of individual particles count, though substantial dilutions involved require special precautions to avoid artifacts, especially in the nano range. However, neither DLS nor SAXS deal with Doxil^®^ morphology, nor do they show distributions based on measuring large number of individual liposomes which is expected to give a much better information on the drug-product and its performance.

Precise measurements of liposome morphological features by cryo-TEM and SAXS, accompanied by the particle count provided by NTA, allowed us to quantitatively evaluate the liposome lipid bilayer and PEG corona volumes as well as the doxorubicin-sulfate nano-rod crystal structure and dimensions.

Cryo-TEM is a frequent subject to criticism for its limitation that the images obtained are two-dimensional projections of the 3D objects that are being studied. Three-dimensional scattering techniques, such as X-ray, DLS etc., on the other hand, have other limitations: they are statistical by their nature thus providing only the average data rather than analyzing individual particles, which limits their ability to fully understand real distributions and their implications to the particles’ performance. In addition, they operate with mathematical models based on various assumptions.

As mentioned above, cryo-TEM characterizes individual particles rather than the particles population as an entity. Therefore, it raises issues such as what sampling size (by number of NPs) may be considered as representative of the whole population, and how many particles should be analyzed for the results to be of statistical significance. This study also addresses these highly important issues.

### 3.1. Population Size Required to Achieve Meaningful Distributions

Recent American Society for Testing and Materials (ASTM) guidance [27] calls to “record enough images at the appropriate magnification to constitute a suitable data set”. However, it is unspecific as to what “enough” or “suitable” means. Neither does it set any acceptance criteria.

To address this question and to characterize the variability of the method as well as to try and define which population could be considered sufficiently “representative”, statistical analysis was performed on the data covering a total of 5011 detected liposomes:

- Subpopulations consisting of 10, 25, 50, 100, 200, 500, 1000 and 2500 particles were randomly picked from the whole population (N = 5011). This operation was repeated 100 times for each subpopulation.
- The average values and standard deviation (SD) of each subpopulation were calculated.
- The difference between the average value obtained with each subpopulation and corresponding value obtained with the entire population was calculated.
- The average difference for each subpopulation size was calculated.

This methodology was chosen as the particle size distribution is asymmetrical (see Figure 1) and cannot be described as normal.

**Figure 2:**
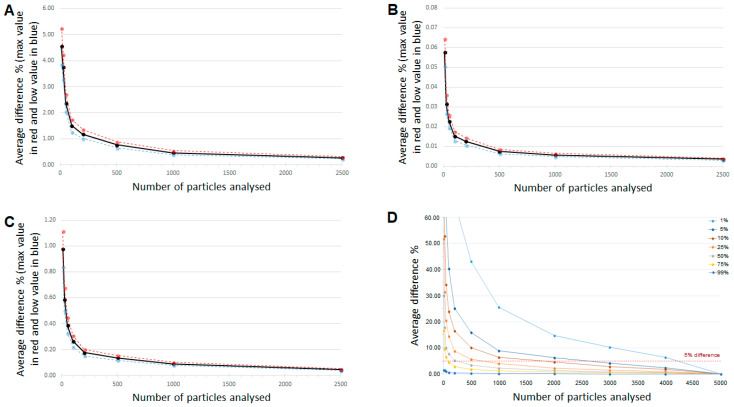
(**A**) Average difference in diameter, (**B**) circularity and (**C**) aspect ratio between data obtained for population of a certain size to that obtained for the reference (5011 liposomes) as a function of population size. (**D**) Average difference between data obtained for each sample size to that obtained from the whole dataset as a function of sample size for various “filled ratios”.

Dependence of the average difference between the liposome diameter, circularity, and aspect ratio measured with the entire population and a subpopulation as a function of the subpopulation size is presented in (Figure 2). The average differences along with the confidence intervals (shown as dashed lines) are plotted. It is demonstrated that results variability and correspondent confidence intervals expand dramatically for both, liposome diameter and aspect ratio, when the population size drops below 500 particles.

Liposome morphology parameters such as “Filled Ratio” (the ratio between liposomes with or without doxorubicin sulfate crystal inside) and “Lamellarity” (“multilamellar” means liposomes with two or more membranes, see Section 3.6) are presented as binary data, i.e., “1” represents a filled or unilamellar vesicle, whereas “0” represents an empty or multilamellar vesicle.

A more general approach was applied for binary parameters. Seven randomized datasets containing 5000 particles were generated with 1%, 5% 10%, 25%, 50%, 75% and 99% of filled (or unilamellar) particles, respectively. From each dataset, subpopulations of 10, 25, 50, 100, 200, 500, 1000, 2000, 3000 and 4000 particles were randomly sampled, and the ratios of filled to empty particles were calculated. This process was iterated 100 times and the average difference between each subpopulation size to the whole dataset was plotted (Figure 2D). It is possible to see that this difference decreases as the filled ratio (or % of unilamellar particles) increases. More specifically, if taking 5% difference as the acceptance criterion, a sample size of more than 4000 particles is required for a significant result for “filled ratio” (or % of unilamellar particles) of below 50%. On the other hand, when the estimated “filled ratio” (or % of unilamellar particles) is above 50%, sample size of 500 particles is enough.

Aspect ratio=Minor axisMajor axis

(vice versa ratio, referred to as elongatedness, is traditionally used).

Adequacy of greater than 500 particles population was further demonstrated by an interlaboratory experiment, in which the cryo-TEM images of the same sample of Doxil^®^-like liposomes were acquired by two laboratories equipped with different instruments (see below). Image processing was also performed independently. Impressive conformance of the three datasets thus generated may be noticed (Table 1).

### 3.2. Liposome Size and Size Distribution

To better understand how measurements based on diffusion rate models and the concept of hydrodynamic diameter, such as DLS, NTA and centrifugal techniques, correlate with data obtained from analysis of cryo-TEM images, standard polystyrene particles were studied.

On one hand, excellent agreement was demonstrated between the intensity-based “z-average” diameter of 76.0 ± 0.2 nm measured by DLS and 75.8 nm calculated from diffusion coefficient determined by ultracentrifugation. On the other hand, smaller (though with higher SD) average diameter of 62 ± 12 nm derived from 544 particles detected in 19 cryo-TEM images (Supplementary Materials Figure S1) conforms to the average 62 ± 0.1 nm of the number-mean diameter measured by DLS.

DLS results are normally presented in three different ways, based on intensity, volume, or number distribution statistics.

The intensity-based Z-average size (also known as the “cumulants mean”) is the most direct and, therefore, considered the most important and stable measure recommended for quality control purposes. It is independent on additional nanoparticle characteristics such as density and refractive index. However, unless the sample size distribution is monomodal, it can hardly be correlated with the direct size measurements provided by electron microscopy techniques such as cryo-TEM. This is mainly due to the bias related to the fact that the intensity of scattered light is proportional to the sixth power of the particle diameter and, therefore, even small fraction of larger particles may have significant impact on the measured mean size [28].

Volume-based distributions are biased in a lesser extent, as the contribution of a bigger particle would be greater to the third power of the diameter ratio compared to the smaller particle.

This is exemplified with measurements of standard polystyrene NPs:

Number mean = 62.0 ± 0.1 nm

Volume mean = 70.3 ± 0.1 nm 8 nm bias (3rd power involved)

Z-average = 76.0 ± 0.2 nm 14 nm bias (6th power involved)

In an ideally unimodal sample, the three values are supposed to be equal. Significant biases occur in the standard polystyrene NPs, even though the particles are relatively mono-dispersed spheres with a polydispersity index (PDI) of only 0.02.

TEM measurements also confirmed fairly monomodal but rather wide size distribution of standard polystyrene particles with a SPAN $\left(\frac{D_{90}-D_{10}}{D_{50}}\right)$ of ~0.5. DLS measurement produced similar SPAN of ~0.6 with a PDI of 0.02. These data demonstrate poor sensitivity of PDI to distribution broadness and justify the use of SPAN as a better measure of size distribution than PDI.

Table 2 presents data collected for Doxil^®^-like dispersion by two different but related light-scattering techniques (DLS and NTA, in both sizes are calculated from the diffusion coefficient), compared with data obtained from direct size measurements from cryo-TEM images. In-light of the above, it is the number mean measured by DLS that is expected to correlate with the mean size measured from cryo-TEM images analyzed by VAS (Vironova Analyzer Software). Yet, 61 and 56 nm were found, respectively. The origin of this 5 nm bias lies in the difference between the regular polystyrene NPs and PLD particles. In the latter the PEG corona, discussed below, contributes significantly to a larger hydrodynamic diameter. Similar mode value of 63 nm was measured also by NTA, which is tracking individual particles, yet based on hydrodynamic diameter and assumption of spherical NP structure.

Accounting for the PEG corona “invisible” in TEM, the “number” mean of DLS and the mode mean of NTA are best correlating with the cryo-TEM.

In cryo-TEM the thickness of the vitreous ice may have an impact on the liposome size determination, as large liposomes tend to accumulate in areas with thick ice and be excluded from thin ice areas. This is especially apparent when using grids with pure holey carbon film, where on-grid size sorting due to formation of a vitreous ice meniscus is a commonly observed effect [27]. In-order to mitigate any such bias on the liposome size determination, holey carbon grids overlaid with a thin film of continuous carbon were used. In addition, a semi-randomized image acquisition regimen was used, and several remote grid locations were included in each analysis.

### 3.3. Circularity Versus Aspect Ratio

Liposomes tend to be formed with shapes having a different extent of “roundness”. In their planar projection, there are several common ways to express closeness to an ideal round sphere outline:

$$Circularity=\sqrt{\frac{4\pi \times Area}{Perimeter^{2}}}$$

expresses both closeness of the shape to a circle as well as “roughness” of its perimeter;

Solidity= AreaConvex hull area

is the function of an overall shape concavity;

Convexity=Convex hull perimeterPerimeter

is mainly the measurement of “roughness” of the shape edge;

Convexity=Convex hull perimeterPerimeter

vice versa ratio (which is also called elongatedness) is traditionally usedas a straightforward relationship between the length and the width of the particle projection.

Each one of the above measurements best serves different shape types [29]. Thus, for instance, both convexity and solidity are unable to distinguish between circle and ellipse, whereas aspect ratio would not differentiate between symmetrical outlines such as square or triangle. Circularity, on the other hand, is the most universal measure, capable of differentiating any deviation from the round shape. Yet, as demonstrated in Figure S2 (in the Supplementary Materials), particularly in the case of smooth elliptical shapes, such as liposomes, the aspect ratio measurement is much more sensitive to such deviations.

On average, rounded to three significant digits, circularity of 1.00 ± 0.00, but aspect ratio of 1.07 ± 0.04 were measured for the Doxil^®^-like liposomal sample used in this study, demonstrating the impact of the intra-liposome doxorubicin-sulfate nano-rod crystal on liposome morphology. In parallel, both parameters were equal to 1.00 ± 0.00 for “empty” liposomes without the doxorubicin sulfate crystal inside (measured on 971 particles).

Figure S3 (in the Supplementary Materials) presents the corresponding distributions. It is clearly demonstrated that in assemblies such as Doxil^®^ the aspect ratio (elongatedness) is much more informative than the circularity, implying that the latter is not sensitive enough to evaluate the shape of this kind of particles.

Here we also show that cryo-TEM is a powerful tool in determining NP size and morphology distribution and therefore that it is highly useful for the optimization of drug products based on NPs.

**Figure 3:**
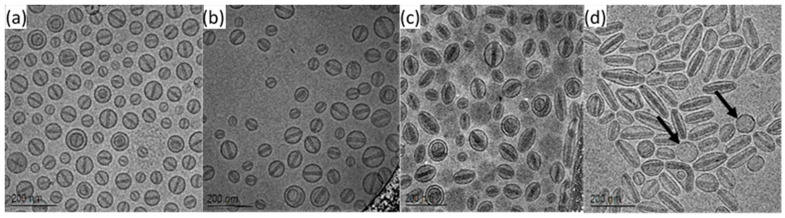
Liposome elongatedness as a function of drug loading: (**a**) 1 mg/mL, (**b**) 2 mg/mL, (**c**) 3 mg/mL and (**d**) 4 mg/mL—black arrows point at spherical empty liposomes.

**Figure 4:**
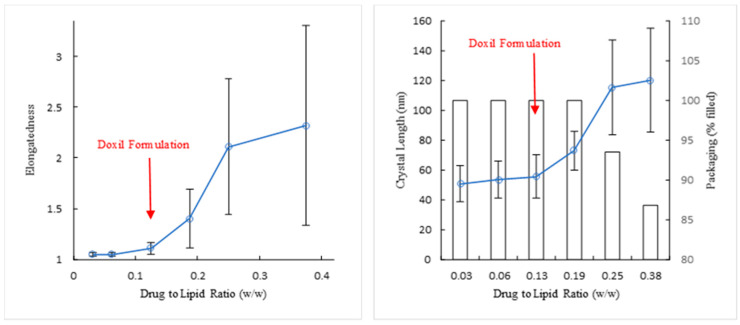
Elongatedness of liposomes (**left**), crystal length and packaging (**right**) as a function of the drug to lipid ratio.

This is demonstrated clearly in Figure 3 and Figure 4 which present the images (Figure 3) and morphology distributions measured for liposome elongatedness, % of liposomes filled with doxorubicin-sulfate nano-rod crystals, and length of these intra-liposomal crystals (Figure 4) of PLD of different drug-to-lipid ratio prepared using liposomes of the same size, lipid concentration, and lipid composition, but remotely loaded with different amounts of doxorubicin. Figure 4, left and right, show good correlation between the doxorubicin-sulfate crystal length and liposome elongatedness, indicating that the latter is determined to a large extent by the doxorubicin-sulfate nano-rod intra-liposome crystal length. Only cryo-TEM can determine such major structural features. This part of our study confirms that, in fact, Doxil^®^ formulation is optimal with respect to the drug to lipid ratio in terms of maximum “drug cargo” per liposome, on one hand, and least possible liposome shape distortion on the other. These morphological parameters have important implications, both clinically (complement activation [30]) and technologically—distorted liposomes (e.g., Figure 3d) might present a real processability obstacle under sterile filtration. Besides, dramatic rise of variability (Figure 4) in both crystal length and liposome elongatedness indicates that greater than optimal (Doxil^®^) drug-load leads to highly inhomogeneous population. Significant decrease of the filled ratio, or, in other words, increase in the count of empty liposomes (Figure 4 right) is another interesting phenomenon. The force induced by formation of too long drug crystal cannot be accommodated by the liposome, the membrane ruptures, ammonium and pH transmembrane gradients are collapsed leading to the formation of an “empty” spherical liposome (Figure 3d).

### 3.4. Lipid Bilayer Thickness

The liposome membrane thickness in a Doxil^®^-like PLD was measured on either 50 or 100 liposomes as 5.6 ± 0.4 nm and 5.6 ± 0.5 nm, respectively. The PLD membrane is a continuous lipid bilayer of which the “liposome forming lipid”, HSPC, arranges the lipid bilayer matrix. As such, its thickness is well known and should be independent on the liposome size. Indeed, in our previous SAXS [6] studies we confirmed the expected bilayer thickness and demonstrated phosphorous head to phosphorus head distance of 4.8 ± 0.1 nm. The direct observations by cryo-TEM and the SAXS [6] measurements are surprisingly alike (Table 3). For cryo-TEM, the thickness of the liposome membrane was determined by analyzing the radial density profile of the particles using the Vironova Analyzer Software. The highest second derivate for the electron density gradient at the inner and outer leaflet was used to define the inner and outer edge of the membrane.

The bilayer thickness measured by cryo-TEM is slightly greater, which may be attributed to the choline part contribution of the phosphatidylcholine head group. Note that the PEG part of both leaflets of the lipid bilayer is invisible by the cryo-TEM due to insufficient contrast.

**Figure 5:**
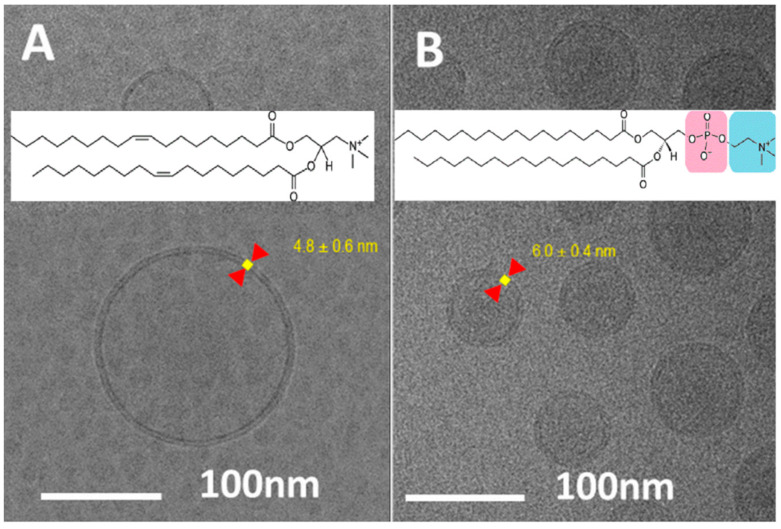
Cryo-TEM images of empty (drug free) liposomes based on DOTAP (**A**) vs. HSPC (**B**). Schematic representation of DOTAP and HSPC is shown on images (**A**,**B**), respectively.

To test the effect of lipid head group size on membrane thickness determination by cryo-TEM, we analyzed and compared images of drug-free “empty” Doxil^®^-like liposomes and liposomes based on DOTAP (1,2-dioleoyl-3-trimethylammonium propane) instead of HSPC. Since DOTAP lacks the phosphate, its head group is smaller compared to HSPC. Besides, DOTAP with unsaturated oleyl acyl-chains, forms less rigid membrane above its T_m_ (compared to the saturated mostly stearoyl acyl-chains of HSPC, below its T_m_) [31]. The combination of these two differences suggests smaller thickness of DOTAP based liposome lipid bilayers, which was confirmed by the cryo-TEM images (Figure 5). The membrane thickness was measured as 4.8 ± 0.6 nm for DOTAP versus 6.0 ± 0.4 nm for HSPC based liposomes. This difference of about 12 Angstrom (1.2 nm), although four times the size of tetrahedral phosphate (highlighted by magenta in Figure 5B HSPC molecule) is explained by the variance in head-groups size of DOTAP and HSPC and demonstrates the ability of cryo-TEM to account for such differences. Yet, it is to be said that these measurements should be improved as currently they depend primarily on the pixel size of about 0.4 nm, like the obtained SD.

Additional important observation is that the membrane thickness of PLD is not affected by the crystal inside the liposome. This confirms that there is no direct interaction between the doxorubicin sulfate nano-rod crystal and the lipid bilayer, which is in agreement with what was previously found from SAXS [6] and DSC [22] comparing Doxil-like liposomes with and without remotely loaded doxorubicin.

### 3.5. Polyethylene Glycol (PEG) Layer Thickness

When PEGylated liposomes were firstly introduced in the nineties [11,12,13,15,16,17,18,19,32,33], the dimension of the PEG (2000 Da) moiety of the MPEG-DSPE used could not be measured directly. The resolution of SAXS used back then was not good enough [20] and, in cryo-TEM, the PEG due to its huge amount of bound water [11] does not give enough contrast to be visualized [20].

The PEG extension from the lipid bilayer was firstly theoretically calculated. Configuration of grafted PEG undergoes “mushroom–brush” transition depending on its concentration at the liposome surface. Up to 4 mol.% of grafted PEG exhibits “mushroom” conformation in which neighboring PEG coils hardly interact in the membrane plane. In the case of more than 8 mol.% PEG, neighboring PEG chains push against each other, and as a result, extend further out from the surface on which they are grafted, leading to the full “brush” conformation. Between 4 and 8 mol.% MPEG-DSPE (2000 Da) it is the region of transition between fully “mushroom” to fully “brush” PEG conformations [34]. Current scaling models [35,36] enable calculating the extension of PEG from the lipid surface of the liposomes.

For the “mushroom” regime, PEG extension length from the surface is given [36] by:

(1) Lmushroom≈a×N35,

where *a* is the monomer length (*a* = 0.35 nm for ethylene glycol) and *N* is the degree of polymerization (*N* = 46 for PEG2000). According to this equation, *L_mushroom_* for PEG2000 is about 3.5 nm.

For the “brush” regime, extension length from the surface is given [36] by:

(2) Lbrush≈a×N35D23,

where *D* is the distance between points of grafted PEG. According to this equation, *L_brush_* for PEG2000 can reach about 10 nm for fully PEGylated micelles.

With increase of MPEG-DSPE concentration above 8 mol.%, the theory predicts the PEG–PEG interaction to perturb the surface and to start a transition process from liposomes to micelles.

There is about 5.0 mol.% of MPEG-DSPE (2000 Da) in Doxil^®^. Based on the above considerations [34], PEG conformation is closer to “mushroom” rather than to “brush”. This is in a good agreement with recent SAXS measurements of 4.8 nm, which became feasible due to a combination of improved technology and software [6,21].

**Figure 6:**
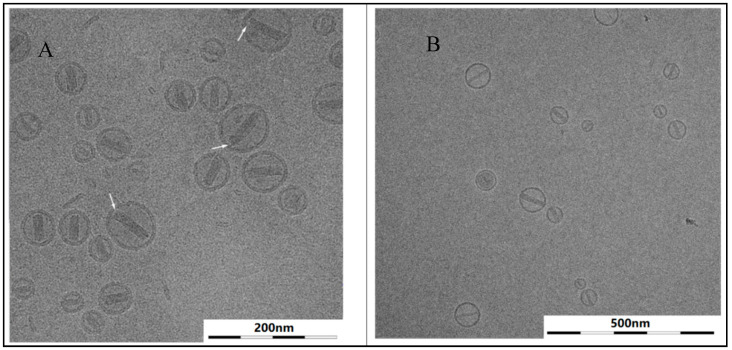
High-resolution cryo-TEM images of liposomes: (**A**) with polyethylene glycol (PEG) (white arrows point at layer gap), (**B**) without PEG.

To date, SAXS was believed to be the only technique capable of direct measurement of the apparent thickness of the PEG corona surrounding the liposome surface. Due to the lack of enough contrast, the PEG corona is invisible in the cryo-TEM images. This obscurity may, nonetheless, be utilized for straightforward measurements in the case of a Doxil^®^-like liposomal product: as clearly visualized by high-resolution (HR) cryo-TEM, the crystal of doxorubicin sulfate grows longitudinally as far as it starts stretching the membrane. Yet, it never directly “touches” the membrane (Figure 6A) but rather some nearly constant gap is always observed at sufficiently high resolution. Since it is the crystal which is directly responsible for the “stretched” elongated projections, at least in those orientations of the liposomes where the elongatedness is clearly seen, the gap may only be explained by the presence of the PEG layer. On the contrary, in liposomes made of HSPC and cholesterol without DSPE–PEG constituent in the membrane (Figure 6B), there are multiple occurrences where the drug crystal seems to be closer attached to the liposome membrane. It is noteworthy that “no-gap” appearances are also feasible in some projections of liposomes with PEG due to a certain orientations of liposome particles in the vitreous ice (see Figure S4 in the Supplementary Materials).

There are two possibilities to measure the gap between the crystal and the membrane in the cryo-TEM images: directly, or indirectly by subtraction of the crystal length (L, nm) and twice the membrane thickness (h, nm) from the outside liposome diameter (D, nm—major axis) and dividing it by 2.

For the indirect method, the crystal length and outside liposome diameter of 100 liposomes were measured, generating the average dimensions of 47.9 nm and 63.1 nm, respectively.

Thus, using the average values the following gap was obtained:

(3) $$\frac{\left(D-2\times h-L\right)}{2}=\;\frac{63.1\;\mathrm{nm}-2\times 5.6\;\mathrm{nm}-47.9\;\mathrm{nm}}{2}=2.0\;\mathrm{nm}$$

SAXS measurements [6] of the same sample revealed 3.6 ± 1.2 nm thickness of the PEG layer on the liposome internal membrane surface (facing the intra-liposome aqueous phase) and 4.8 ± 1.2 nm on the external surface of the liposome membrane facing the extra-liposome aqueous medium (Table 3). This difference in PEG length was attributed to the effect of the curvature on the PEG chains packing. SAXS measurement, as a statistical method, yields overall mean values for all the surfaces, inside and outside, in contrast to direct imaging by cryo-TEM. Measurement of the gap in the cryo-TEM images produces the thickness of the PEG layer on the inside membrane surface compressed (by the intra-liposome nano-rod crystal) in the direction the liposome is stretched along. However, the mean “inside” SAXS value of 3.6 nm accounts for both parts of the PEG layer inside the liposome, compressed and uncompressed. Thus, it is expected to obtain thicker inside PEG layer by SAXS than by cryo-TEM measurements as is indeed the case here. However, the fact that cryo-TEM cannot measure the PEG layer directly remains unaltered.

SAXS measurements from the same work [6] on spherical liposomes, without crystal inside (and thus without compressed PEG layer) revealed a 4.1 ± 1.2 nm PEG layer thickness on the inside membrane surface and 4.6 ± 1.2 nm outside the liposome. This difference may stem from the averaging nature of the scattering technique in addition to the possible contribution of the different curvature as was suggested before [6].

The “gap” and elongatedness, combined, provide a general 3D perspective of the liposome orientation in the vitrified layer based on the following considerations:

It was shown that drug free (empty) unilamellar nanoliposomes are perfectly round (elongatedness = 1.000). In the “filled” liposomes, every occurrence where the crystal “touch” the membrane is detected by the liposome elongatedness greater than 1.000.

Various orientations of elongated liposomes in the vitrified layer are manifested as different apparent gaps between the crystal edge and the membrane measured on the planar projection (Figure S4 in the Supplementary Materials):

(4) $$\mathrm{Gap}\;=\frac{1}{2}\times \left(\mathrm{Minor}\;\mathrm{Axis}\;-\;Membrane\;Thickness\;-\;Crystal\;Width\;-\;Crystal\;Length\times \cot\varphi \;\right),$$

where: φ—polar angle of projection; in the particular case of the crystal edge “face-up” projection, φ = 90°, and

(5) $$\mathrm{Gap}\;=\;\frac{1}{2}\times \left(\mathrm{Minor}\;\mathrm{Axis}\;-\;Membrane\;Thickness\;-\;Crystal\;Width\right)$$

The “face-up” projections may also be confirmed by the elongatedness close to 1.

Depending on projection, the apparent crystal length will change as:

Apparent Crystal Length = Crystal Length ×cosφ+Crystal Width ×sinφ

In general, the idea is that the larger is the gap, the larger is the polar angle, provided elongatedness larger than 1 is confirmed.

### 3.6. Lamellarity

**Figure 7:**
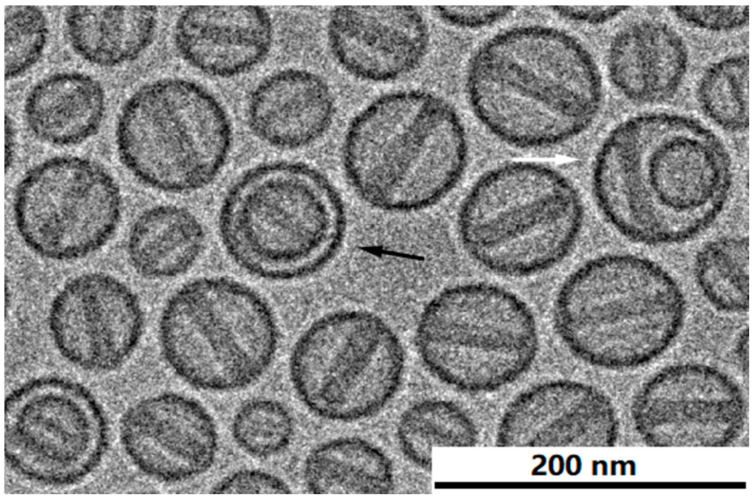
Cryo-TEM image with concentric (black arrow) and eccentric (white arrow) bi-lamellar liposomes.

Two types of multilamellar particles were detected in a Doxil^®^-like dispersion (Figure 7). To discuss the impact of these peculiar formations, it is necessary to understand the mechanism of doxorubicin remote loading [37]. “Blank” (empty, without drug) liposomes are primarily formed to contain substantial amount of ammonium sulfate. As an ionic species it cannot permeate the membrane in either direction. Removal of the salt from the extra-liposomal environment by means such as dialysis, results in significant concentration difference of ammonium and sulfate ions across the liposome membrane. This results in creation of the transmembrane ammonium and pH gradients which are responsible for literally “pumping” the drug inwards against the doxorubicin concentration gradient. Therefore, this mechanism, is referred to as “remote active loading”. It is followed by crystallization, once doxorubicin encounters sulfate ions in the intra-liposome aqueous phase. This whole process is driven by the ammonium gradient [10].

In the case of the multilamellar structure, the gradient is only built up across the outer interface, while the concentration of ammonium sulfate remains even in all the inner aqueous compartments. Therefore, remote loading of doxorubicin into such a structure progresses first into the intermittent cavity if there is enough space. The crystal then shapes to fit the available cavity space while pushing the inner vesicle aside. Such an eccentric structure is clearly visible in Figure 7 (white arrow). Otherwise, where there is not enough space between the inner and outer membranes, the loading process continues further inwards, and the crystal grows in the innermost compartment, forming a concentric structure as pointed by the black arrow in Figure 7. As a matter of fact, this concentricity may only occur when PEG layers occupy the interstitial compartment entirely.

### 3.7. The Morphology of Intra-Liposome Doxorubicin Sulfate Nano-Rod Crystal

**Figure 8:**
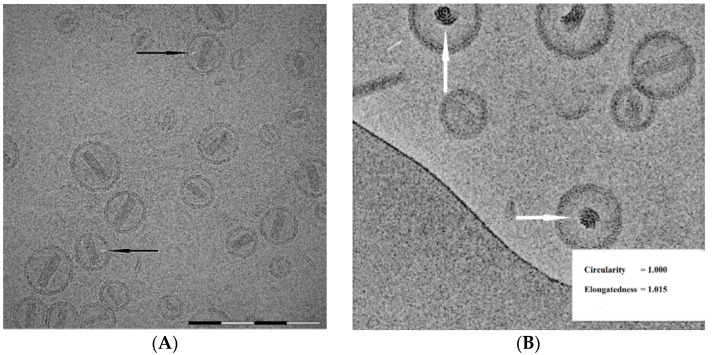
Fine fiber structure visualized in longitudinally (**A**) and transversally (**B**) oriented liposomes. Black arrows show twisted crystal bundles. White arrows present crystal filaments “face-up” view. Elongatedness of 1.015 indicates that the liposome is stretched by the crystal. The scalebar on the left is 200 nm.

High resolution cryo-TEM allows visualization of fine structural details of the doxorubicin sulfate crystal inside the liposome. The nano-rod crystals consist of a somewhat twisted bundle of filaments (Figure 8). When good images are acquired, the crystal dimensions are relatively easy to measure and the number of filaments across the crystal can be counted. On average, the length of doxorubicin sulfate crystals was evaluated as 48 ± 9 nm measured on 100 liposomes.

Table 4 presents the distribution of 100 liposomes as a function of crystal length (in nm) and number of filaments across the bundle.

It is worth noting, that as cryo-TEM describes morphology in two dimensions, it is difficult to assess the actual number of fibers per an intra-liposomal doxorubicin sulfate rod-shape nanocrystal. We assume that the number of fibers per nanocrystal is larger than reported here. It could be well seen on the “face-up” oriented nanocrystal (Figure 8B). Other approaches such as high resolution cryo-electron tomography have-to be applied in-order to get the correct numbers. Still the information presented here shows right trends.

As expected, the larger liposomes (Figure 6) contain longer crystals. It is interesting to note that the domain size of doxorubicin sulfate filaments was estimated by SAXS to be 15 ± 2 nm [6] which may indicate the crystal length has a stepwise increment with a fixed value rather than continuous longitudinal growth.

The width (diameter) of doxorubicin sulfate nano-rod crystals was evaluated as 16 ± 2 nm measured on 500 liposomes. Table 5 presents the dependence of doxorubicin-sulfate nano-rod crystal width (nm) on the number of filaments detected in its cross-section. It appears that the crystal grows with a constant increment of about 2.5 ± 0.1 nm, which, in fact is its latitudinal lattice constant [6].

The width of doxorubicin sulfate nano-rod crystal measured by cryo-TEM (16 ± 2 nm) agrees with that measured by SAXS (17 ± 4 nm, [6]). A lower standard deviation for the data obtained by direct measurement using cryo-TEM may imply a better precision of this method compared with the scattering technique.

Unfortunately, SAXS sensitivity was found insufficient to analyze the intra-liposomal crystal’s fine structure in the intraliposomal aqueous phase. However, SAXS data available for bulk doxorubicin sulfate crystals [6] indicate a hexagonal lattice with constants a = b = 3.18 nm and c = 2.02 nm. Bulk crystals are evidently denser compared to the filament rods found in liposomes.

## 4. Conclusions

As the most important “take-home” lesson, our study demonstrated that on many structural and morphological variables (size, shape, membrane-thickness, intra-liposome drug physical shape) cryo-TEM, accompanied by the state-of-the-art image-processing means, is at least as informative as and complementary to X-ray scattering techniques. However, while the latter gives the ensemble statistical averages of the studied variable population-as-a-whole, the cryo-TEM provides distributions based on the measured variable of individual liposomes from which a distribution of individual liposomes and its average are calculated. In the case of a drug that forms an assembly in the intra-liposome aqueous phase, the cryo-TEM also allows to obtain the size distribution and morphologies of these assemblies.

We found that an automated image-processing of a population greater than 500 particles is sufficient to generate reproducible size and morphological distributions.

It was demonstrated that quantitative analysis of liposome morphology and shape distributions provides a powerful tool for optimization of the nano-drug formulation, both in terms of the drug cargo, processability and possible clinical implications.

Quantitative cryo-TEM in some parameters related to the lipid membrane bilayer and doxorubicin nano-rod crystal dimensions is correlative and, in some parameters, complementary to SAXS. New insights into the fine structure of this crystal also make this method complementary to SAXS.

High-resolution cryo-TEM enables evaluation of the PEG layer thickness inside the liposome, even though it is technically “invisible”.

However, so far, due to the lack of sufficient contrast cryo-TEM is unable to give direct data on the dimension and structure of the liposomes’ PEG corona, which currently can only be directly achieved by SAXS [12].

Since cryo-TEM keeps improving, it may become one of the techniques of choice to characterize nano-drugs and similar complex nanoparticles.

## Figures and Tables

**Table 1:** Size and morphology of Doxil^®^-like liposomes: inter-laboratory comparison.

Images Acquired	Images Processed	No of Images	No of Liposomes	Diameter (nm)	Unilamellar (%)	Elongatedness
Lab A	Lab A	11	923	59 ± 13	99	1.10 ± 0.07
Lab A	Lab B	6	561	53 ± 11	99	1.15 ± 0.08
Lab B	Lab A	3	690	57 ± 11	98	1.08 ± 0.06
Lab B	Lab B	5	5011	56 ± 11	98	1.07 ± 0.04

**Table 2:** Size and size-distribution of Doxil-like Liposomes: comparing measurements by dynamic light scattering (DLS) and nano-tracking analysis (NTA) and cryo-TEM.

Method	Liposome Diameter, nm	Percentiles, nm			SPAN
		D10	D50	D90	
DLS	z-Average 76 ± 1	56	78	110	0.7
	Number mean 61 ± 2	45	59	82	0.6
	Volume mean 70 ± 2	49	67	98	0.7
NTA	Mean 73 Mode 63	42	69	104	0.9
Cryo-TEM	Mean 56 ±11	42	54	71	0.5

**Table 3:** Morphology data of the same Doxil^®^-like sample comparing small-angle X-ray scattering (SAXS) [6] and Cryo-TEM.

Dimensions (nm)	SAXS *	Cryo-TEM	Comments
Membrane Thickness	4.8 ± 0.1	5.6 ± 0.5	SAXS estimates phosphorous-to-phosphorous, while TEM accounts for choline-to-choline distance
PEG Layer	3.5 ± 1.2 inside 4.8 ± 1.2 outside	~2.0	Indirect measurement of the gap between the nanocrystal and the membrane by cryo-TEM
Crystal Length	-	48 ± 9	
Crystal Domain Size	15 ± 2	-	
Crystal Width	17 ± 4	16 ± 2	
Crystal Filament	-	~2.5	

* From Schilt et al. [6].

**Table 4:** Distribution of crystal length of doxorubicin Sulfate per Number of Fibers in the Crystal (measured on 100 liposomes).

%	100	23	60	16	1
Number of Fibers	–	5	6	7	8
Crystal Length (nm)	48 ± 9	46 ± 10	49 ± 9	46 ± 6	61 ± n/a

**Table 5:** Crystal Width of intra-liposome doxorubicin sulfate as a function of the number of fibers in the crystal (measured on 500 liposomes).

Number of Fibers	Overall	4	5	6	7	8
Crystal Width (nm)	16 ± 2	11.0 ± 0.7	13.4 ± 0.6	16.0 ± 0.5	18.5 ± 0.5	21.0 ± 0.5
Increment (nm)	–	–	2.4	2.6	2.5	2.5

## Data Availability

Not applicable.

## Supplementary Materials

The following are available online at https://www.mdpi.com/1999-4923/13/1/123/s1, Figure S1: Representative original (A) and processed (B) cryo-TEM Images of standard polystyrene particles, (C) size distribution histogram. Figure S2: Circularity versus aspect ratio of an elliptical shape. Figure S3: A and B: aspect ratio and circularity of Doxil^®-^like liposomes, respectively; C: aspect ratio of “empty” liposomes. Figure S4: Apparent length and the distance between the crystal edge and the liposome membrane.
