# Supplementary material for: Quantitative Cryo-TEM Reveals New Structural Details of Doxil-Like PEGylated Liposomal Doxorubicin Formulation
Source: Pharmaceutics. 2021 Jan 19;13(1):123. doi: 10.3390/pharmaceutics13010123 (PMC7835975; doi:10.3390/pharmaceutics13010123)
Supplement: Supplementary file 1 [file pharmaceutics-13-00123-s001.pdf]

pharmaceutics

Supplementary Materials: Quantitative Cryo-TEM Reveals New Structural Details of Doxil-Like PEGylated Liposomal Doxorubicin Formulation

Rickard Nordström, Lin Zhu, Johan Härmark, Yael Levi-Kalisman, Erez Koren, Yechezkel Barenholz, Genia Levinton and Dima Shamrakov

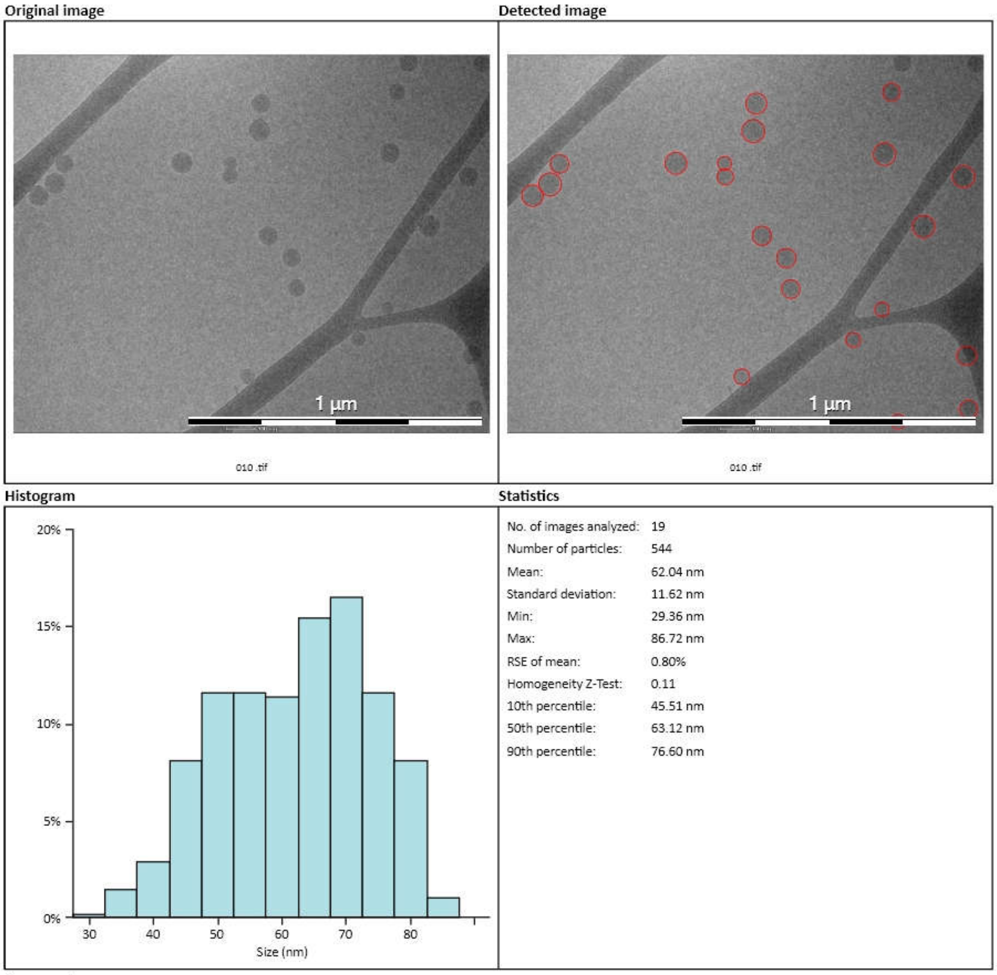

Figure S1. Representative original (**A**) and processed (**B**) cryo-TEM Images of standard polystyrene particles, (**C**) size distribution histogram.

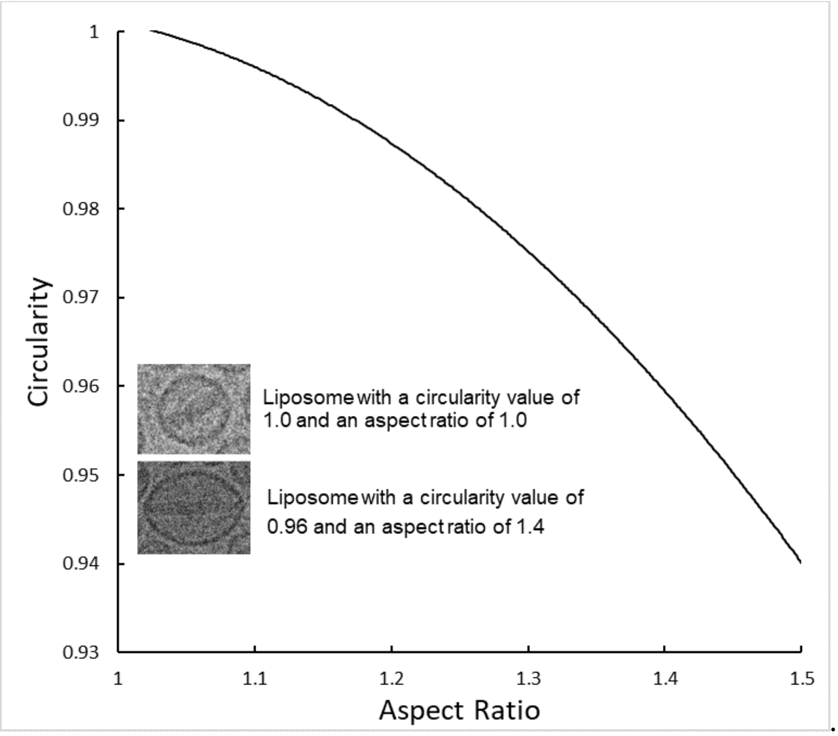

Figure 2. Circularity versus aspect ratio of an elliptical shape.

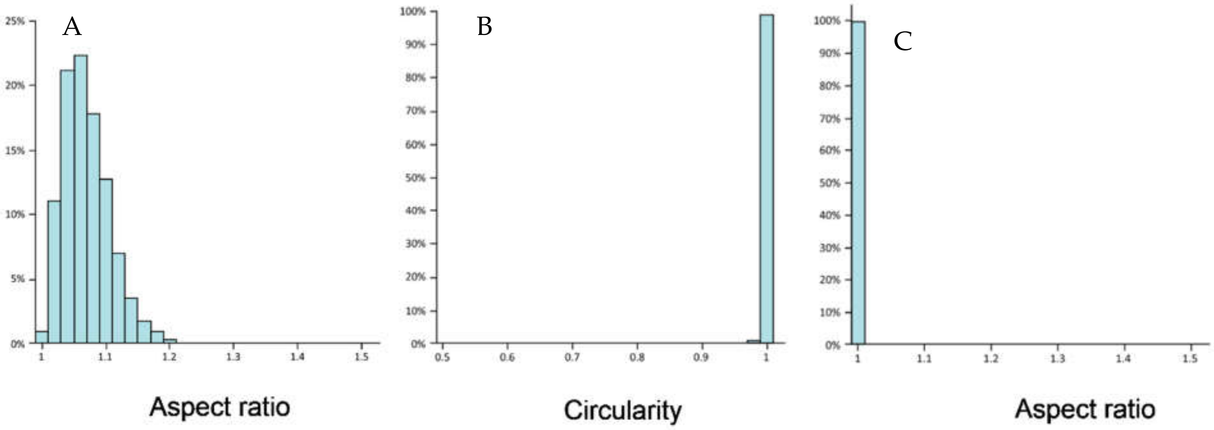

Figure S3. (**A**) and (**B**): aspect ratio and circularity of Doxil®-like liposomes, respectively; (**C**): aspect ratio of "empty" liposomes.

$$\text{Apparent Gap} = \frac{1}{2} \times (\text{Minor Axis} - \mathit{Membrane\ Thickness} - \mathit{Crystal\ Width} - \mathit{Crystal\ Length} \times \cot\varphi)$$

$$\text{Apparent Crystal Length} = \text{Crystal Length} \times \cos\varphi + \mathit{Crystal\ Width} \times \sin\varphi$$

where: φ – polar angle of the projection;

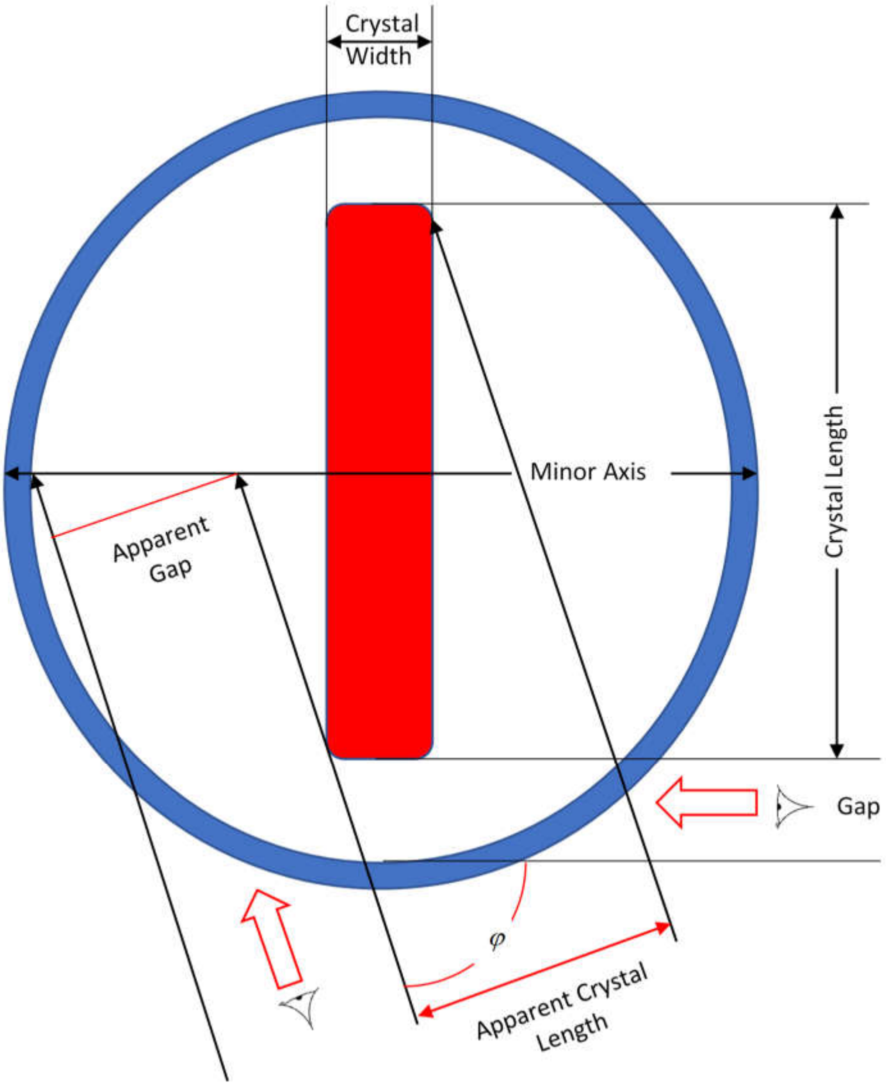

Figure S4. Apparent length and the distance between the crystal edge and the liposome membrane.
